# Stress-related multisystem dysregulation during adolescence predicts mental health symptoms in young adulthood

**DOI:** 10.1017/S0033291725102377

**Published:** 2025-11-04

**Authors:** Sabine Finlay, Oyelola Adegboye, Donna Rudd, Brett McDermott, Zoltan Sarnyai

**Affiliations:** 1 Laboratory of Psychiatric Neuroscience, James Cook University, Townsville, QLD, Australia; 2 Australian Institute of Tropical Health and Medicine, James Cook University, Townsville, QLD, Australia; 3 College of Medicine and Dentistry, James Cook University, Townsville, QLD, Australia; 4 Margaret Roderick Centre for Mental Health Research, James Cook University, Townsville, QLD, Australia; 5 Menzies, School of Health Research, Royal Darwin Hospital Campus, Casuarina, NT, Australia; 6 Child and Adolescent Mental Health Service, Hobart, TAS, Australia; 7 Department of Psychiatry, University of Tasmania, Hobart, TAS, Australia

**Keywords:** adolescence, allostatic load, depression, psychosis, stress

## Abstract

**Background:**

Adolescence is a critical period for brain maturation, influenced by stress and hormonal changes. Chronic stress can lead to increased allostatic load (AL), a cumulative measure of multisystem dysregulation, and insulin resistance (IR), both of which are linked to mental health disorders. We hypothesized that heightened AL and IR during adolescence (age 17) would predict the emergence of mood and psychotic symptoms in young adults.

**Methods:**

This study used data from the Avon Longitudinal Study of Parents and Children, a population cohort from Bristol, United Kingdom.

**Results:**

Our results showed that elevated AL at age 17 was significantly associated with the development of mood disorder symptoms (MDS) and psychotic disorder symptoms (PDS) and the co-occurrence of mood and psychotic disorder symptoms (MPDS) at age 24 (*p* < 0.001). Mean AL increased progressively across these symptom groups, indicating a dose–response relationship between physiological dysregulation and mental health burden (MDS = 3.67, PDS = 3.89, and MPDS = 4.03). We also observed that IR was significantly elevated in the MDS, PDS, and MPDS groups compared to healthy controls (HCs). IR was most prevalent in the PDS group, suggesting a possible association between metabolic dysfunction and psychosis risk.

**Conclusion:**

This study demonstrated that multisystem dysregulation in late adolescence precedes the onset of mood and psychotic symptoms in early adulthood. These results support the use of AL and metabolic markers as early indicators of psychiatric vulnerability and highlight the potential for early intervention targeting systemic dysregulation to prevent the onset of mental health disorders.

## Introduction

Childhood and adolescence are sensitive periods of brain development, during which the body and the brain are subjected to a myriad of different exposures from the external environment. Many of those exposures present as adversity, including both psychosocial (maltreatment, such as abuse and neglect, and family-related adversities) and biological (suboptimal nutrition, pollution, toxins, etc.). Early adversity is particularly impactful on healthy brain developmental trajectory during sensitive periods, such as the period between the ages of 5 and 12, between the ages of 12 and 18, and between 12 and early adulthood, when socioemotional characteristics, executive functions, and self-referential processes develop and mature, respectively (C. A. Nelson, Sullivan, & Valdes, 2025). Key brain circuits involving the hippocampus, amygdala, and prefrontal cortex underlie these functions. Their altered developmental patterns can give rise to the development of mood (amygdala and hippocampus) and psychotic (prefrontal cortex) symptoms and eventually to diagnostic psychiatric conditions, such as major depressive disorder and psychosis spectrum disorders (Bora, Yucel, & Pantelis, 2009; Hastings, Parsey, Oquendo, Arango, & Mann, 2004; MacQueen et al., 2003; Shin & Liberzon, 2010). Therefore, it is of key importance to better understand how biological mechanisms may mediate the effects of early adversity, any environmental exposure with negative neurodevelopmental and behavioral impact, on the development of mental health disorders to facilitate early identification of high-risk individuals and to apply appropriate interventions in time.

Early adverse experiences of diverse nature represent deviations from expected inputs from the environment and, therefore, constitute stress for the organism and activate the canonical biological stress response. This includes the rapid activation of the sympathetic nervous system (SNS), resulting in the release of adrenaline from the adrenal medulla and the noradrenaline-mediated effects of the sympathetic nerve terminals throughout the body, followed by the activation of the hypothalamic–pituitary–adrenal (HPA) axis that results in the release of the glucocorticoid hormone cortisol. In addition, immune-inflammatory molecules, such as pro-inflammatory cytokines, are also activated to exert both systemic and brain effects (McEwen, 1998b; McEwen & Stellar, 1993). This acute activation of stress response helps the organism to rapidly adapt to the deviations to the expected norms by redistributing blood, and therefore oxygen and energy sources, to tissues essential for the survival (SNS), to mobilize extra energy sources (HPA axis), and to prepare for fighting potentially invading pathogens (pro-inflammatory cytokines), a coordinated biological process conceptualized as *allostasis*, maintaining stability through change (McEwen, 1998a; McEwen & Stellar, 1993). However, as in the case of early adversity, deviations from the expected environmental inputs continue to be present, not allowing the appropriate negative feedback processes to normalize the activity of these biological systems, each producing chemicals with profound effects on fundamental biological processes, with considerable bioenergetic cost over time (McEwen, 1998b, 2007; McEwen & Stellar, 1993). The cost of chronic exposure to fluctuating or continually heightened neural and neuroendocrine responses resulting from repeated or chronic environmental challenges is referred to as *allostatic load* (AL) (McEwen, 1998b, 2007). AL reflects the cumulative effects of experiences in daily life that involve ordinary events, major challenges, such as early adversity and life events, and physiological consequences of health-damaging behaviors (poor sleep, lack of physical activity, and unhealthy diet) (Suvarna et al., 2020). When these environmental challenges, repeated deviations from the expected norms, exceed the individual’s biological ability to cope, *allostatic overload* develops, resulting in processes affecting the functional integrity of the body as well as the brain (McEwen, 2007). AL and allostatic overload can be captured by measuring the cumulative impact of stress over time through biomarkers representing the multisystemic nature of the concerted physiological response. This measure, termed *AL index*, is constructed by identifying, and summing up, the individual biomarkers from the key stress-responsive physiological systems, including the SNS, neuroendocrine, cardiovascular, metabolic, and immune system, that are in either too high or too low, depending on which confers a greater physiological risk for health (McEwen, 1998a; Seeman, Singer, Rowe, Horwitz, & McEwen, 1997).

Chronic stress-related increases in the AL index have been linked to a variety of systemic and mental health disorders. For example, elevated AL has been shown to contribute, in the longer term, to the development of cardiovascular diseases (Gillespie et al., 2019; Guidi, Lucente, Sonino, & Fava, 2020), type II diabetes (Carlsson, Andreasson, & Wändell, 2011; Steptoe et al., 2014), musculoskeletal disorders (Mori et al., 2014; Smith, Maloney, Falkenberg, Dimulescu, & Rajeevan, 2009), cancer (Parente, Hale, & Palermo, 2013; Ruini, Offidani, & Vescovelli, 2015), and metabolic syndrome (Almadi, Cathers, & Chow, 2013; Osei, Block, & Wippert, 2022). AL index has been found to be elevated in psychiatric disorders and associated with psychotic symptoms (Berger, Juster, et al., 2018; Nugent, Chiappelli, Rowland, & Hong, 2015; Piotrowski et al., 2019) and depression (Arnaldo, Corcoran, Friston, & Ramstead, 2022). With relevance to children and adolescents, a significant association has been shown between AL and cognitive functions, such as attention and working memory deficits (D’Amico, Amestoy, & Fiocco, 2020; Evans & Schamberg, 2009; Rogosch, Dackis, & Cicchetti, 2011). Furthermore, elevated AL has been associated with mental health symptoms (MHS) in children and adolescents (Nelson, Sheeber, Pfeifer, & Allen, 2020; Rogosch et al., 2011).

Beyond the established associations between elevated AL index and already existing physical and mental health conditions, longitudinal studies have shown that elevated AL also predicts later development of impaired functioning, morbidity, and mortality (Guidi et al., 2020; Seeman, McEwen, Rowe, & Singer, 2001; Seeman et al., 1997). Similarly, elevated AL has been associated with the later emergence of mental health disorders, such as mood and psychotic disorders, and impaired cognition (Booth et al., 2015; Friedman, Karlamangla, Gruenewald, Koretz, & Seeman, 2015; Gou et al., 2025; Kapczinski et al., 2008; Li, Yan, & Li, 2024; Perlman, Cogo-Moreira, Wu, Herrmann, & Swardfager, 2022). Recent attention has been paid to linking AL in childhood and adolescence to later health consequences through longitudinal studies, as summarized in a recent systematic review. This review concludes that greater AL is associated with poorer health outcomes in both clinical and non-clinical pediatric populations, with possible long-term effects (Lucente & Guidi, 2023). Specifically, elevated AL was shown to be associated with mental health issues, including depression (Nelson et al., 2020; Rogosch et al., 2011). We have shown that although the AL index in childhood was not directly associated with the later development of psychotic disorder symptoms (PDS) and mood disorder symptoms (MDS) in a longitudinal birth cohort, females in the higher tertiles of the AL index measured at 9 years of age had an elevated risk of MHS as young adults (Finlay, Adegboye, McDermott, Rudd, & Sarnyai, 2025). This finding raises the possibility that further accumulation of adverse events during adolescence and their impact on systemic stress biology, and hence on AL, might be required to give rise to MHS in early adulthood.

Puberty and following adolescence are sensitive periods for brain development, especially in the domains of socioemotional, executive, and self-referential processes (Nelson et al., 2025). With regard to the changes in the HPA axis activity, research demonstrates that puberty is a period of excessive HPA axis plasticity and that the effects of early adversity on cortisol regulation change with pubertal maturation. This concept is formalized in the ‘pubertal stress recalibration hypothesis’, which suggests that for the HPA axis, puberty may reopen the system and functionally create a second sensitive period (DePasquale, Herzberg, & Gunnar, 2021; Gunnar, DePasquale, Reid, Donzella, & Miller, 2019). Furthermore, the adolescent brain is uniquely sensitive to stress, as demonstrated by differential, stress-induced structural changes in the key brain structures of emotional regulation and executive functions, such as the amygdala, hippocampus, and prefrontal cortex, respectively, potentially giving rise to stress-related psychiatric disorders that emerge post-adolescence (Romeo, 2017). Since most psychiatric disorders have their onset before the age of 25 (Kessler et al., 2005), it is imperative to better understand how early biological vulnerability may contribute to long-term psychopathology.

**Figure 1.**
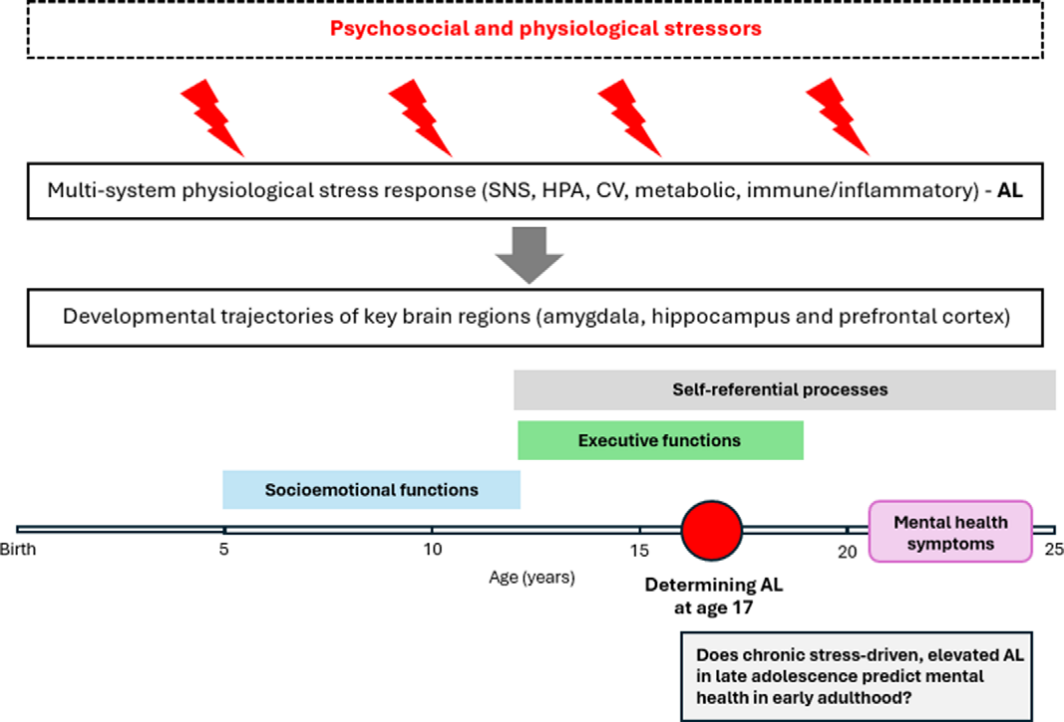
A graphic representation showing how chronic stress may result in the overactivation of stress response, leading to an impact on the structural development of key brain regions, which can contribute to psychopathology. Our working model posits that psychosocial and physiological stressors during childhood and adolescence activate a multisystem physiological stress response characterized by the activation of stress mediators, such as cortisol, norepinephrine, and pro-inflammatory cytokines. These mediators, when produced in excess or chronically over a long period, driven by continuing stress and adversity, impact the structural development of brain areas key to emotional regulation, higher cognitive functions, and behavioral control, and therefore, they can contribute to ensuing psychopathology. Based on this, we hypothesized that elevated AL, the physiological hallmark of chronic stress, in late adolescence, predicts the emergence of mental health symptoms in early adulthood.

Our working model (Figure 1) is based on the accumulated knowledge that chronic stress (early adversity, physiological stressors, and adolescent psychosocial stress) results in the overactivation of the same physiological processes that initially help adaptation (*allostasis*) and lead to elevated AL, which the established measure of stress-related multisystem dysregulation, the AL index, can capture.

Furthermore, the physiological stress mediators, such as cortisol, norepinephrine, and pro-inflammatory cytokines, have been shown to impact the structural development of brain areas key in emotional regulation, higher cognitive functions, and behavioral control, and therefore, they can contribute to ensuing psychopathology. Based on this, we hypothesized that elevated AL, the physiological hallmark of chronic stress, in late adolescence predicts the emergence of MHS in early adulthood. To test this hypothesis, we first examine whether the concerted activation of multiple physiological systems, including cardiovascular, lipid metabolism, glucose metabolism, immune, and anthropometric systems at 17 years, as well as the AL index, was associated with the emergence of MHS before age 25 using data from the Avon Longitudinal Study of Parents and Children (ALSPAC). If such an association can be established, we then aim to explore whether the individual physiological systems included in the AL index are differently linked with the emergence of MHS to identify key systemic processes that may drive later psychopathology.

## Methods

### Description of cohort and sample selection

Pregnant women (*n* = 14,541) resident in Avon, United Kingdom, with expected delivery dates from April 1, 1991, to December 31, 1992, were invited to take part in the study and sent a questionnaire to complete (Boyd et al., 2013; Fraser et al., 2013; Northstone et al., 2019). Of these initial pregnancies, there was a total of 14,062 live births, and of those, 13,988 children were alive at 1 year of age. When the oldest children were approximately 7 years old, an attempt was made to increase the initial sample with eligible cases who had failed to join the study initially. The total sample, including the later enrolment phases, is 14,701 children (alive at 1 year of age). The enrolled mothers and their children have been followed continuously since the study’s inception and continue to be monitored today. Study data were collected and managed using REDCap electronic data capture tools hosted at the University of Bristol (Harris et al., 2009). REDCap is a secure, web-based software platform that supports data capture for research studies. Please note that the study website contains details of all the data that are available through a fully searchable data dictionary and variable search tool. Full cohort details appear elsewhere (Finlay et al., 2023). Our sample group consisted of participants with data on 10–13 biomarkers and available data on MHS before age 26 (*n* = 1479).

### Laboratory measures

For this study, data from 13 biomarkers were obtained to index the functioning of the cardiovascular, immune, and metabolic systems. For details regarding biomarker measurements, collection, and assay procedures, see Finlay et al. (2023). We included biomarkers measuring cardiovascular functioning, including systolic and diastolic blood pressure and heart rate. Measures of immune functioning included C-reactive protein (CRP). Measures of lipid metabolism included cholesterol, high-density lipoprotein (HDL), low-density lipoprotein (LDL), triglycerides, very-low-density lipoprotein (VLDL), and adiponectin. Glucose metabolism was assessed using fasting glucose and insulin levels. For the anthropometric system, we included body mass index (BMI) (Supplementary Table 1).

#### Homeostatic model assessment for insulin resistance (HOMA-IR)

HOMA-IR analysis was calculated by fasting glucose (mmol/L) and insulin (mIU/L) levels at age 17 years using the following formula: (glucose × insulin)/22.5. A HOMA score between 0 and 1.9 was considered ‘normal’, 2–2.9 was considered ‘early insulin resistance’ (EIR), and above three was considered ‘insulin resistance’ (IR).

### Cutoff points for biomarkers

Each biomarker result was calculated using sample quartiles as cutoffs (i.e., upper or lower quartiles depending on whether low or high values confer a greater risk for health). For all participants, each biomarker was assigned a score of 0 (*normal value*) or 1 (*either above the 75th percentile or below the 25th percentile*), depending on the biomarker’s risk ranking. The 25th and 75th percentiles were determined based on the data distribution in participants who did not develop MHS (healthy controls [HCs]).

### The allostatic load index calculation

The AL index was calculated at age 17 using the ‘group allostatic load index’ approach (R. P. Juster, McEwen, & Lupien, 2010). Like the above scores, each biomarker result uses sample quartiles as cutoffs (i.e., upper or lower quartiles depending on whether low or high values confer a greater risk for health). Scores were then summed together across all biomarkers for a total score ranging from 0 to 13. Higher total scores indicate greater AL or physiological dysregulation because of chronic stress.

### Mental health symptoms

Schizophrenia was assessed using the item ‘ever been diagnosed with schizophrenia’ with a dichotomous answer (yes/no) at the age of 22 years. Psychotic symptoms were assessed using the item ‘psychotic symptoms (ever)’ with a dichotomous answer (yes/no) at the age of 24 years. The Psychosis-Like Symptom Interview (PLIKSi) was conducted by trained psychology graduates in assessment clinics. It was coded according to the definitions and rating rules for the Schedules for Clinical Assessment in Neuropsychiatry. Psychotic episodes (PEs) covered the three main domains of positive psychotic symptoms: hallucinations (visual and auditory), delusions (spied on, persecuted, thoughts read, reference, control, and grandiosity), and thought interference (insertion, withdrawal, and broadcasting). After cross-questioning, interviewers rated PEs as *not present*, *suspected*, or *definitely present.* Cases of PEs in this study were defined as individuals with *definite* PEs.

Mood disorder was assessed using the survey item ‘ever been diagnosed with depression’ and ‘ever been diagnosed with bipolar disorder’, with a dichotomous answer at the age of 22 years. Furthermore, depressive symptoms were assessed at the age of 25 years using the 13-item Short Mood and Feelings Questionnaire (SMFQ), designed for the assessment of depression symptoms in children and adolescents (Angold, Costello, Messer, & Pickles, 1995). Each item is rated on a three-point Likert scale (0 = not true, 1 = sometimes true, and 2 = true), resulting in a total score between 0 and 26, with higher scores indicating greater depressive symptoms. In this study, scores of 12 or higher were classified as indicative of clinically relevant depressive symptoms (Thapar & McGuffin, 1998). Individuals with both MDS and PDS will be referred to as those with ‘MPDS’ henceforth.

### Confounders

The sex of the participants, mother’s occupation (or social class), and mother’s age at delivery were treated as confounders. Sex was recorded at birth and treated as a binary variable (male or female). Mother’s occupation was recorded at birth using the UK Office of National Statistics’ socioeconomic classification system (class I = professional; class II = managerial and technical workers; class IIIa = skilled non-manual occupations; class IIIb = skilled manual occupations; class IV = partly skilled occupations; class V = unskilled occupations). From this point forward, this will be referred to as ‘social class’.

### Statistical analysis

All analyses were performed using SPSS v23 and *R version 4.0.3.* Descriptive analysis was performed and stratified by ‘MHS’. Differences in prevalence between categorical variables were tested using chi-square tests. In case of small sample sizes (<5), Fisher’s exact test was used. In contrast, differences in prevalence between several independent groups were tested appropriately using analysis of variance (ANOVA) or the Kruskal–Wallis test. Linear regression was used to examine the relationship between HOMA grouping (normal, EIR, and IR) and HCs, MDS, PDS, and MPDS groups. The assumptions of linear regression were subsequently checked. To examine the inter-relationship among the five biological systems (cardiovascular, lipid metabolism, immune, glucose metabolism, and anthropometric), gender, social class, mother’s age at birth, and AL index, we conducted an inter-correlation analysis using Pearson’s correlation coefficient. Before conducting the correlation analysis, we checked the assumptions of normality for continuous variables using the Shapiro–Wilk test. Correlation coefficients were interpreted using the guidelines proposed by Cohen (2013), where *r* values ranging from 0.10 to 0.29 represent a small effect size, 0.30–0.49 represent a medium effect size, and > 0.5 represent a large effect size. Lastly, we used logistic regression to estimate odds ratios (ORs) and 95% confidence intervals (CIs) to assess the association between AL at age 17 years and the development of MDS, PDS, and MPDS. To determine statistical significance, we used a significance level (alpha) of 0.05 for all tests.

## Results

### Descriptive statistics for the total sample, controls, and cases

We included 1479 participants in this study, with more than ten biomarkers collected and reported. Of these, 542 individuals had any MHS (36.64%).

There were more females than males in the MHS group (65% versus 35%). Mother’s age at delivery did not differ between the HCs and the MHS group (29.91 versus 29.88; p = 0.92), and neither did the percentage distribution of individuals in the different social classes (high = 50% versus 46%, middle = 44% vs 45%, and low = 6% and 8.4%, p = 0.21, respectively).

### Contribution of different biological systems in the allostatic load index

**Figure 2.**
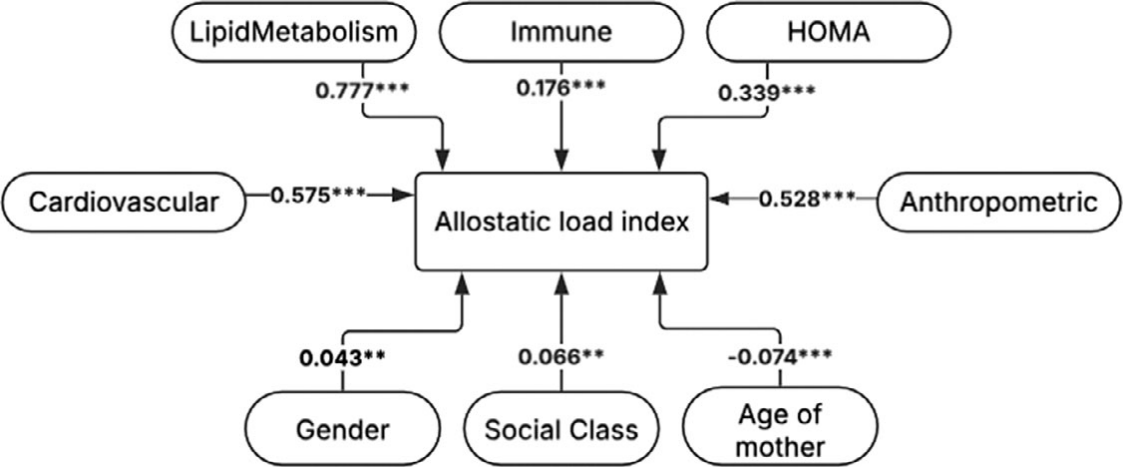
Correlation coefficient values indicate the independent variable’s positive or negative influence on the dependent variable (AL index at age 17). Significance is shown in the figure as ‘***’ is p < 0.001 and ‘**’ is p = 0.005.

By using the four biological systems investigated, ‘lipid metabolism’, ‘cardiovascular’, ‘immune’, and ‘HOMA’, we created the system-specific AL indices. In Figure 2, a positive correlation coefficient value indicates that as the value of one variable (such as mother’s age at delivery) increases, the other variable (e.g., AL index) also increases. We observed significant correlations between various biological systems formulating the AL index. For example, ‘lipid metabolism’ exhibited a stronger positive correlation with the AL index compared to ‘cardiovascular system’ (**r** = 0.777 vs 0.575, respectively).

#### The allostatic load index in MDS, PDS, and MPDS

**Figure 3.**
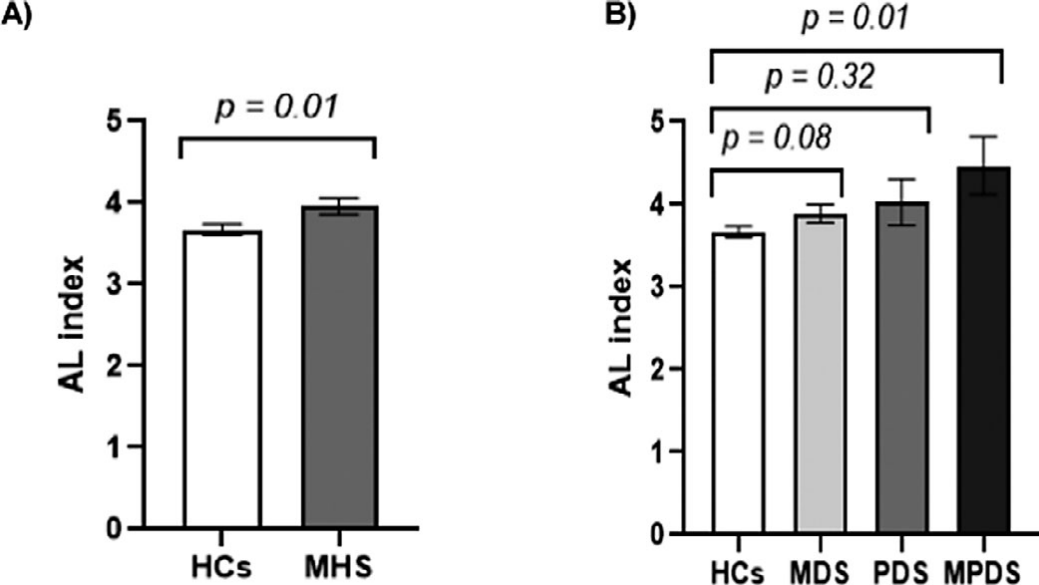
(a) Mean AL index at age 17 for healthy controls (HCs) vs individuals with any mental health symptoms (MHS). (b) Mean AL index at age 17 for HCs, individuals with mood disorder symptoms (MDS), individuals with psychotic disorder symptoms (PDS), and individuals with both mood and psychotic disorder symptoms (MPDS). Significant values are shown on the graphs.

The AL index at age 17 was significantly higher in individuals who developed MHS by 25 years of age than the HCs (3.96 versus 3.67, *p* = 0.01) (Figure 3a). In Figure 3b, it can be observed that the AL index is higher at age 17 in individuals who developed either MDS, PDS, or MPDS compared to the HCs and that the AL index is proportionally increasing with the severity of the disorder (from MDS to MPDS). A sub-analysis showed that the odds of developing MDS, PDS, and MPDS in relation to AL were only significantly increased for MPDS (*p =* 0.03). In contrast, associations with MDS and PDS were not significant (*p =* 0.12 and 0.21, respectively) (Supplementary Table 2).

### Separating MDS, PDS, and MPDS

Of the 542 individuals with MHS, 410 participants (75.6%) reported having MDS, 87 participants (16%) reported having PDS, and 45 individuals (8.3%) reported having MPDS (Supplementary Table 3). More females than males developed MDS (67%) before 25 years of age (*p* = 0.01). A similar, but not significant, number was observed for the MPDS group, with 73% females developing this (*p =* 0.07). In contrast, more males than females developed PDS (51%) before 25 years of age (*p* = 0.05).

**Figure 4.**
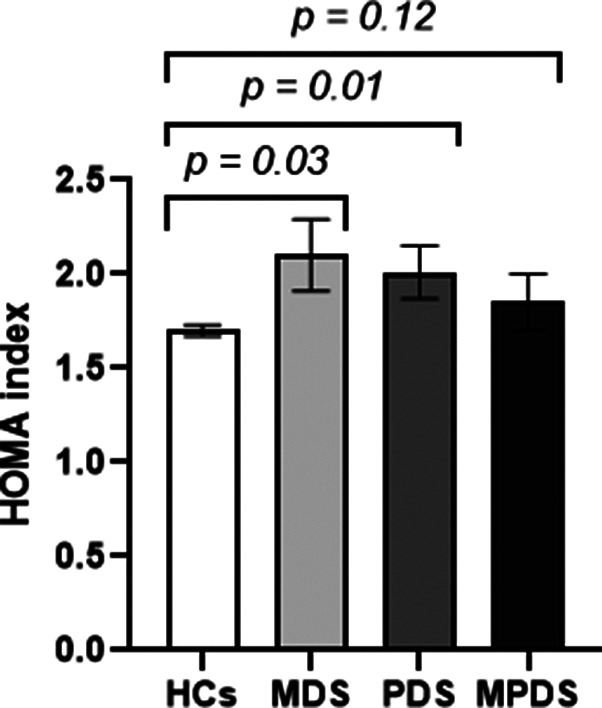
HOMA index between healthy controls (HCs), mood disorder symptoms (MDS), psychotic disorder symptoms (PDS), and mood and psychotic disorder symptoms (MPDS). Significance is shown on the graph.

Mother’s age at delivery did not differ between the HCs, and MDS, PDS or MPDS (29.91 ± 0.14 versus 30.02 ± 0.22 (*p* = 0.71) versus 29.27 ± 0.48 (*p* = 0.29) versus 29.82 ± 0.65 (*p* = 0.98), respectively). Similarly, the percentage of individuals in the three social class groups (low, middle, and high) was not different between the HCs (low = 6%, middle = 44%, and high = 50%), and those with MDS (low = 9.3%, middle = 42.7%, and high = 48%; *p* = 0.14), PDS (low = 8.2%, middle = 49%, and high = 42%; *p* = 0.31) and MPDS (low = 0%, middle = 62%, and high = 38%; *p* = 0.08). HOMA scores were significantly higher in individuals who developed MDS or PDS, compared to the HCs (*p =* 0.03 and *p =* 0.01, respectively) (Figure 4).

The cardiovascular biomarkers were the highest for individuals with MPDS among all groups (MPDS mean = 0.93 ± 0.14 versus PDS mean = 0.89 ± 0.10 versus MDS mean = 0.80 ± 0.04 versus HC mean = 0.78 ± 0.02) (Supplementary Table 3).

The lipid biomarkers were the highest for individuals with PDS among all groups; however, there was no significance between the MPDS, PDS, and MDS groups versus the HCs (MPDS mean = 2.33 ± 0.25 versus PDS mean = 2.48 ± 0.16 versus MDS mean = 2.11 ± 0.07 versus HC mean = 2.06 ± 0.04), *p =* 0.53, 0.12, 0.62, respectively) (Supplementary Table 3).

The immune biomarker CRP was the highest for individuals with MPDS among all groups. However, there was no significance between the MPDS, PDS, and MDS versus the HCs (MPDS mean = 2.61 ± 1.12 versus PDS mean = 1.41 ± 0.32 versus MDS mean = 1.36 ± 0.21 versus HC mean = 1.46 ± 0.14); *p* = 0.13, 0.77, 0.21, respectively) (Supplementary Table 3).

The overall metabolic biomarker BMI was the highest for individuals with MDS, closely followed by individuals with MPDS (MPDS mean = 22.62 ± 0.54 versus PDS mean = 22.54 ± 0.44 versus MDS mean = 22.63 ± 0.18 versus HC mean = 22.29 ± 0.11).

### Homeostatic model assessment for insulin resistance (HOMA-IR)

**Figure 5.**
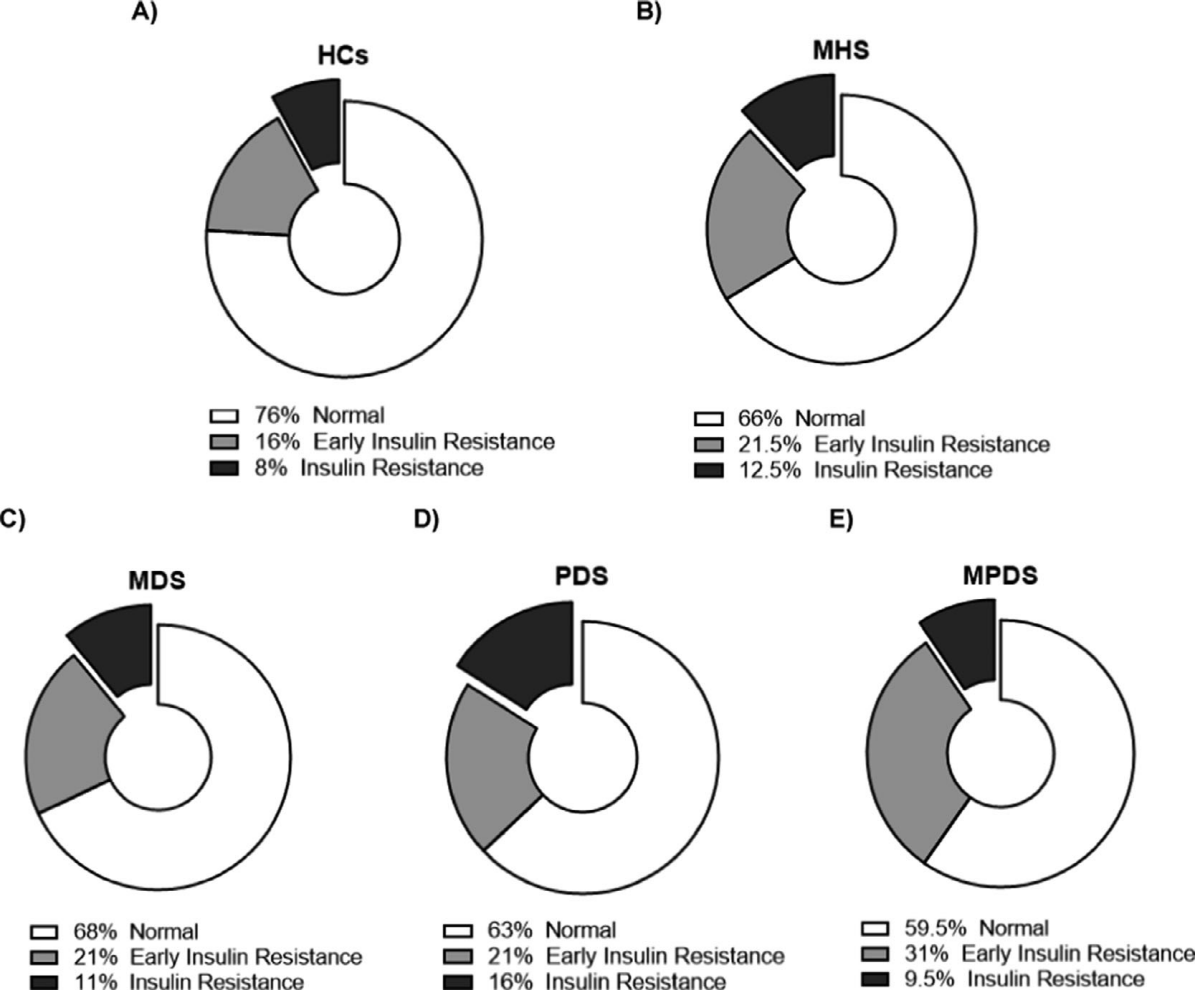
Percentage of individuals with HOMA levels indicating ‘normal’ (white), ‘early insulin resistance’ (light grey), and ‘insulin resistance’ (dark grey) within the groups. (a) HCs, ‘Healthy controls’; (b) MHS, ‘mental health symptoms’. (c) MDS, ‘mood disorder symptoms’; (d) PDS, ‘psychotic disorder symptoms’ and (e) MPDS, ‘mood and psychotic disorder symptoms’.

Analysis of the HOMA-IR showed a significant difference in the number of individuals with ‘normal’ HOMA levels, HOMA levels indicating ‘EIR’, and HOMA levels indicating ‘IR’ between the HCs and those with MHS (*p <* 0.001) (Figure 5a,b). The results from the three MHS groups, MDP, PDS, and MPDS, indicated a significant difference between the HCs and the MDS and PDS groups (*p =* 0.01, 0.01, respectively), but no difference between the HCs and the MPDS group (*p =* 0.10) (Figure 5c-e). Using linear regression, we analyzed the three levels ‘normal’, ‘EIR’, and ‘IR’ separately between the HCs, and MDS, PDS, and MPDS. There was a significant difference in the distribution of HOMA categories between individuals with PDS versus the HCs (*p* = 0.01). Compared to the HCs, individuals with PDS had higher rates of IR (16% vs 8%) and EIR (21% vs 16%). Although the MDS group had slightly more individuals in the EIR group (21% vs 16%) and the IR group (11% versus 8%) compared to the HCs, these differences were not significant (*p =* 0.21 and 0.09). Individuals with MPDS were significantly more likely to fall into the IR and EIR categories compared to HCs (Fisher’s exact test, *p* = 0.028). Specifically, 31% had EIR compared to 16% among the HCs, and 9.5% had IR compared to 8% among the HCs.

## Discussion

This study investigated whether late adolescence allostatic multisystem dysregulation influenced by a diverse variety of past stressors is linked to MHS in early adulthood in a large population-based prospective birth cohort. We found that: (1) elevated AL at age 17 was significantly associated with the co-occurrence of MPDS in young adulthood; (2) late adolescence AL gradually increased in relation to MHS severity in early adulthood from MDS to PDS to MPDS; (3) the HOMA index at age 17 was significantly elevated in individuals who developed either MDS or PDS compared to the HCs; and (4) IR was significantly more prevalent in the MHS group compared to the HCs. Furthermore, IR was the highest in the PDS group.

This is the first study to investigate the link between late adolescence multisystem dysregulation and MHS in early adulthood.

Previous studies have investigated the AL index in patients who are already diagnosed with psychotic and mood disorders, such as schizophrenia (Berger, Lavoie, et al., 2018; Nugent, Chiappelli, Rowland, & Hong, 2015; Piotrowski et al., 2020; Savransky et al., 2018), bipolar disorder (Dargel et al., 2020; Juster, Sasseville, Giguère, Consortium, & Lupien, 2018), and major depressive disorder (Honkalampi et al., 2021; Kobrosly, van Wijngaarden, Seplaki, Cory-Slechta, & Moynihan, 2014) and found significantly increased AL compared to controls. Recent studies have investigated the immune biomarkers IL-6 and CRP in childhood (age 9) in the same ALSPAC study and found that increased IL-6, but not CRP, was associated with an increased risk of PDS and MDS in adulthood (Chu et al., 2019; Khandaker, Pearson, Zammit, Lewis, & Jones, 2014; Perry, Zammit, Jones, & Khandaker, 2021). Another study by Perry et al. (2018) found that IR at the age of 9 significantly increases the risk of developing PEs at age 18. Our recent study on AL in 9-year-olds did not find an overall association between childhood AL and the development of MHS in early adulthood, but it identified that females in the highest AL index tertile had an elevated risk of MHS in young adults in the same longitudinal birth cohort (Finlay et al., 2025). However, our present results indicate that individuals with MHS in early adulthood already had a higher mean AL at 17 years of age compared to the HCs. Interestingly, mean AL at 17 years gradually increased with the severity and complexity of the symptoms in adulthood (MDS = 3.89, PDS = 4.03, and MPDS = 4.47).

Although observational studies implicate metabolic dysfunction as a risk factor for psychiatric disorders, the possibility of reverse causation cannot be ruled out. Mendelian randomization studies have provided mixed support, indicating that the genetic liability for depression or schizophrenia may causally influence inflammatory and metabolic biomarkers (Perry, Burgess, et al., 2021; Zhang, Chen, Yin, Wang, & Peng, 2021). Perry, Burgess, et al. (2021) conducted a bidirectional study and found evidence that suggests schizophrenia may causally influence IR and inflammation. Similarly, the study by Zhang et al. (2021) found that metabolic changes could also be a consequence, as well as a cause, of depressive disorder. These results highlight a possible bidirectional relationship between metabolic dysfunction and psychiatric disorders.

Looking at the results for each biological system, individuals who developed MPDS in adulthood had dysregulation in all but the lipid metabolism system. This trend could also be observed in the cumulative AL score, which was higher for the MPDS group than for the MDS, PDS, and HC groups. This suggests that there might be a continuum in the severity of mental health disorders with regard to their relationship with stress-related multisystem physiological dysregulation. The ‘continuum’ model captures this view by showing that mental health disorders exist along a continuum of severity rather than within discrete diagnostic categories. This perspective acknowledges that psychological symptoms vary in intensity and impact, from mild, subclinical distress to severe, chronic illnesses (Keyes, 2002). One end of the spectrum is characterized by optimal well-being and high functioning, whereas the opposite end involves significant functional limitations, chronicity, and greater clinical needs (Widiger & Samuel, 2005). Individuals may move along the continuum and experience mood fluctuations and other MHS and, according to our present results, may exhibit a continuously increasing multisystem dysregulation.

Our results indicate, in agreement with the findings by Perry, Stochl, et al. (2021) and Perry et al. (2018), that the HOMA score, showing the degree of dysregulation of glucose–insulin homeostasis, at the age of 17 years was significantly higher in individuals who developed MDS, PDS, or MPDS in adulthood compared to the HCs. Interestingly, other systems, when looked at separately, did not show a difference between the HCs and individuals who developed MHS. It is conceivable that the dysregulation of the glucose–insulin homeostasis precedes, and perhaps even drives, the abnormalities in other physiological systems. In fact, there is an emerging view that IR can act as an upstream driver of dysfunction in both immune and lipid metabolic systems (Hotamisligil, 2006; Lumeng & Saltiel, 2011; Samuel & Shulman, 2012). The effect of IR on the brain may account for some of the changes seen in patients with MHS. The bidirectional relationship between metabolic dysregulation and mental health disorders has been intensively studied in recent years (Maksyutynska et al., 2024; Penninx & Lange, 2018; Pillinger et al., 2017; Saxena & Marin-Valencia, 2024). Our findings show that EIR and IR at age 17 were more prevalent in individuals who developed MHS in adulthood compared to the HCs. In line with this finding, Watson et al. (2021) measured IR three times over 9 years in adults without a mental health history and found that a moderate increase in the measures of IR would correspond to an 89% increase in the rate of major depressive disorder over the following 9 years.

Elevated fasting glucose levels have been extensively demonstrated in first-episode, never-medicated individuals with psychotic disorders (Pillinger et al., 2017) and in individuals with depression (Kan et al., 2013). It is not known at present whether such elevation in glucose levels is driven by systemic factors or by impaired brain glucose metabolism and brain energy production through an attempted systemic compensatory effort (Sarnyai & Ben-Shachar, 2024). Several biological mechanisms could link IR to the onset and progression of depression and psychotic disorders. These include IR-driven neuroinflammation (Leonard & Wegener, 2020), central nervous pathways, i.e., brain IR (Kullmann et al., 2016), neurotransmitter dysregulation (de Bartolomeis et al., 2023), and shared genetic vulnerability (Perry et al., 2022). These mechanisms can be linked to one another as it has been shown that IR appears to promote neuroinflammation by impacting neurotransmitters, such as serotonin, dopamine, and norepinephrine, which are closely involved in regulating mood, behavior, and stress (Gold, Licinio, & Pavlatou, 2013; Webb et al., 2017). IR often leads to chronic inflammation in the brain and body, and cytokines such as IL-6, CRP, and TNF-alpha are often elevated in patients with IR (Phosat et al., 2017). These cytokines can cross the blood–brain barrier, affecting brain function and contributing to MHS. Interestingly, our results (Supplementary Table 3) did not indicate a significant dysregulation in the immune system, as measured by possible alterations in CRP levels, in adolescents who later developed MDS, PDS, or MPDS compared to the HCs. In fact, CRP was lower in individuals who later developed MDS or PDS than in the HCs. Other studies have discussed this relationship, but no agreement has been established, as some studies show elevated CRP levels, while others find no difference or, in line with our findings, point toward a negative association (Dessoki et al., 2023; Liu, Ely, Simkovic, Alonso, & Gabbay, 2021). As CRP was the only immune biomarker in this study, it is important to interpret our results cautiously. Given CRP, a key acute-phase protein involved in the inflammatory response and influenced by the actions of cytokines, was the only immune/inflammatory biomarker used, it is impossible to establish whether immune dysregulation was already prevalent at age 17. Elevated IL-6 is often correlated with increased IR, so future studies should include multiple inflammatory biomarkers for a comprehensive understanding of immune changes (Yuan et al., 2024). IR is also associated with the dysregulation of glucocorticoids, such as cortisol, in the HPA axis, which is shown to be associated with depression (Mansur, Brietzke, & McIntyre, 2015), and IR has been shown in psychotic disorders (Meyer & Stahl, 2009; Ray & Khess, 2016). Our findings indicate that 21% of individuals who developed PDS in adulthood had IR at 17 years, compared to 8% of those who did not exhibit any MHS in early adulthood, which translates to an over 2.5 times increased risk of developing PDS in early adulthood, if the individuals already show IR in late adolescence.

In light of the evidence reviewed above, our present results suggest that accumulating stress and adversity during childhood and adolescence may lead to the development of IR early on, possibly contributed by the repeated and long-term exposure to stress-induced increase in glucocorticoids (Kivimäki, Bartolomucci, & Kawachi, 2023; Pervanidou & Chrousos, 2012). The emerging IR, then, in turn, may drive, together with the direct effect of repeated stress, the dysregulation of other major physiological systems, resulting in an increased AL. These factors together may then contribute to changes in adolescent brain development, especially in late-maturing systems, such as the prefrontal cortex, and result in alterations in brain function that can manifest as MHS in later life (Lenart-Bugla et al., 2022). Addressing both IR and elevated AL through metabolic interventions, such as diet and lifestyle changes, early psychotherapy, and careful monitoring of health, may help reduce the risk and likelihood of developing MHS and also improve overall mental and physical health (Finlay, Rudd, McDermott, & Sarnyai, 2022).

### Strengths and limitations

The strengths of this work include the longitudinal design and the inclusion of multiple measures that are informative of multisystemic dysregulation within the AL concept and possible confounders for MDS and PDS. A key limitation of the study is the large number of missing values, a common issue for prospective cohort studies (Pham, Pandis, & White, 2024). Our sample population was significantly small, as we only included participants with more than 10 biomarkers reported to represent AL accurately. Despite this, we found a significant difference in AL for the HCs compared to individuals with MHS, both as a group and separated into individuals with MDS, PDS, and co-occurrence of MPDS. A second limitation is related to the timing of the development of MHS, which were collected and reported before the age of 26 years. PDS and MDS are often diagnosed during adolescence and early adulthood, so several individuals may have been wrongly categorized as ‘HCs’ if they did not display symptoms before the age of 26 (Avenevoli, Swendsen, He, Burstein, & Merikangas, 2015; Tandon, Keshavan, & Nasrallah, 2008). Third, while biomarker cutoffs were derived using the HC group to establish a normative reference for the AL index, we acknowledge this may introduce circularity and reduce generalizability. Future research should consider prospective methods to define biomarker thresholds and include more biomarkers, including stress-related hormonal biomarkers, such as cortisol, and a more extensive profile of immune biomarkers, to accurately examine the neuroendocrine stress system and inflammation prior to MHS, creating a more accurate AL. Lastly, socioeconomic status was based on the mother’s occupation categories at recruitment, which may not reflect broader or more nuanced indicators of socioeconomic status.

## Conclusion

In conclusion, our findings indicate a significant association between biomarker changes representing multisystem dysregulation due to past demands, stress, and trauma on the organism over time during childhood and adolescence and the later emergence of MHS in adulthood. Early systemic metabolic dysregulation may be a significant risk factor in the development of mental health disorders, especially for psychotic disorders. Our results indicate that elevated HOMA, indicating IR, and AL may identify individuals at increased risk of developing mental health disorders in later years, even before displaying relevant symptoms.

## Supporting information

Finlay et al. supplementary material Finlay et al. supplementary material

## Data Availability

ALSPAC data access is through a system of managed open access. The steps below highlight how to apply for access to the data referred to in this article and all other ALSPAC data. The datasets presented in this article are linked to ALSPAC project number B3847. The ALSPAC variable codes highlighted in the dataset descriptions can be used to specify the required variables. Please read the ALSPAC access policy (https://www.bristol.ac.uk/media-library/sites/alspac/documents/researchers/data-access/ALSPAC_Access_Policy.pdf), which describes the process of accessing the data and samples in detail and outlines the costs associated with doing so. You may also find it useful to browse our fully searchable research proposals database (https://proposals.epi.bristol.ac.uk/), which lists all research projects that have been approved since April 2011. Please submit your research proposal for consideration by the ALSPAC Executive Committee. You will receive a response within 10 working days to advise you whether your proposal has been approved. The study website also contains details of all the data that are available through a fully searchable data dictionary: http://www.bristol.ac.uk/alspac/researchers/data-access/data-dictionary/.

## Supplementary material

The supplementary material for this article can be found at http://doi.org/10.1017/S0033291725102377.
