# Supplementary material for: Stress-related multisystem dysregulation during adolescence predicts mental health symptoms in young adulthood
Source: Psychol Med. 2025 Nov 4;55:e334. doi: 10.1017/S0033291725102377 (PMC13058643; doi:10.1017/S0033291725102377)
Supplement: Finlay et al. supplementary material [file S0033291725102377sup001.zip › Supplementary Table 3 (NEW).docx]

**Supplementary Table 3** summarizes participants' descriptive statistics for the individuals with mood disorder symptoms (MDS), psychotic disorder symptoms (PDS), and mood and psychotic disorder symptoms (MPDS). Of the 542 individuals with mental health symptoms, 87 participants (16%) reported PDS at age 24, 410 participants (75.6%) reported MDS and 45 individuals (8.3%) reported having MPDS.

Table 3: Summary statistics of individuals with mood disorder symptoms (MDS), psychotic disorder symptoms (PDS), and mood and psychotic disorder symptoms (MPDS) compared with controls. A significant p-value <=0.05 was bolded.

|  | **Controls  (N = 937)** | **MDS  (N = 410)** | **p-value** | **PDS  (N = 87)** | **p-value** | **MPDS  (N = 45)** | **p-value** |
| --- | --- | --- | --- | --- | --- | --- | --- |
| **Sex** |  |  | **0.01** |  | **0.05** |  | 0.07 |
| Male, n (%) | 376 (40) | 135 (33) |  | 44 (51) |  | 12 (27) |  |
| Female, n (%) | 560 (60) | 275 (67) |  | 43 (49) |  | 33 (73) |  |
| **Age mother** |  |  | 0.71 |  | 0.29 |  | 0.98 |
| Mean | 29.91 | 30.02 |  | 29.27 |  | 29.82 |  |
| Standard Error | 0.14 | 0.22 |  | 0.48 |  | 0.65 |  |
| **Social Class** |  |  | 0.14 |  | 0.31 |  | 0.08 |
| Lower (I and II), n (%) | 46 (6) | 30 (9.3) |  | 6 (8.2) |  | 0 (0) |  |
| Middle (III and IV), n (%) | 334 (44) | 138 (42.7) |  | 36 (49) |  | 21 (62) |  |
| Higher (V and VI), n (%) | 386 (50) | 154 (48) |  | 31 (42) |  | 13 (38) |  |
| **Cardiovascular** |  |  | 0.81 |  | 0.52 |  | 0.31 |
| Mean | 0.78 | 0.80 |  | 0.89 |  | 0.93 |  |
| Standard Error | 0.02 | 0.04 |  | 0.10 |  | 0.14 |  |
| **Lipid metabolism** |  |  | 0.62 |  | 0.12 |  | 0.53 |
| Mean | 2.06 | 2.11 |  | 2.48 |  | 2.33 |  |
| Standard Error | 0.04 | 0.07 |  | 0.16 |  | 0.25 |  |
| **HOMA-IR** |  |  | **0.03** |  | **0.01** |  | 0.10 |
| Mean | 1.70 | 2.10 |  | 2.01 |  | 1.85 |  |
| Standard Error | 0.03 | 0.19 |  | 0.14 |  | 0.15 |  |
| **Immune system** |  |  | 0.21 |  | 0.77 |  | 0.13 |
| Mean | 1.46 | 1.36 |  | 1.41 |  | 2.61 |  |
| Standard Error | 0.14 | 0.13 |  | 0.32 |  | 1.12 |  |
