# Supplementary material for: Stress-related multisystem dysregulation during adolescence predicts mental health symptoms in young adulthood
Source: Psychol Med. 2025 Nov 4;55:e334. doi: 10.1017/S0033291725102377 (PMC13058643; doi:10.1017/S0033291725102377)
Supplement: Finlay et al. supplementary material [file S0033291725102377sup001.zip › Supplementary Table 1 (NEW).docx]

**Supplementary Table 1**

Table 1: Cutoff values based on Healthy Controls (HCs). A value above (or below for HDL) is indicative of dysregulation and will be assigned a score of “1”. Values below (or above for HDL) these cutoff values will be assigned a score of “0” (normal)

| Biomarkers included | Cutoff value based on HCs |
| --- | --- |
| Cardiovascular markers |  |
| *Systolic blood pressure* | >124.5 |
| *Diastolic blood pressure* | >67 |
| *Heart rate* | >71 |
| Immune markers |  |
| *C-reactive protein (CRP)* | >1.27 |
| Lipid metabolism markers |  |
| *Cholesterol* | >4.22 |
| *High-density lipoprotein (HDL)* | <1.48 |
| *Low-density lipoprotein (LDL)* | >2.47 |
| *Triglycerides* | >0.93 |
| *Very-low-density lipoprotein (VLDL)* | >0.42 |
| *Adiponectin* | >14354 |
| Glucose metabolism markers |  |
| *Glucose* | >5.22 |
| *Insulin* | >8.75 |
| Anthropometric markers |  |
| *Body-Mass-Index (BMI)* | >23.83 |
