# Supplementary material for: Stress-related multisystem dysregulation during adolescence predicts mental health symptoms in young adulthood
Source: Psychol Med. 2025 Nov 4;55:e334. doi: 10.1017/S0033291725102377 (PMC13058643; doi:10.1017/S0033291725102377)
Supplement: Finlay et al. supplementary material [file S0033291725102377sup001.zip › Supplementary Table 2 (NEW).docx]

**Supplementary Table 2**

The odds ratio (ORs) between increased AL at age 17 and MDS, PDS, and MPDS developed before age 25 was only significant (*p =* 0.03) for MPDS.

Table 2: Odds ratio (ORs) and 95% Confidence Intervals (CIs) between increased allostatic load (AL) and mood disorder symptoms (MDS), psychotic disorder symptoms (PDS), and mood and psychotic disorder symptoms (MPDS). A significant p-value >=0.05 is highlighted.

|  | Odds Ratio (95% CI) | *p - value* |
| --- | --- | --- |
| MDS | 1.04 (0.99 – 1.09) | 0.12 |
| PDS | 1.06 (0.97 – 1.16) | 0.21 |
| MPDS | 1.13 (1.01 – 1.27) | **0.03** |
